# A single-particle characterization of a mobile Versatile Aerosol Concentration Enrichment System for exposure studies

**DOI:** 10.1186/1743-8977-3-8

**Published:** 2006-05-24

**Authors:** Evelyn J Freney, Mathew R Heal, Robert J Donovan, Nicholas L Mills, Kenneth Donaldson, David E Newby, Paul HB Fokkens, Flemming R Cassee

**Affiliations:** 1 School of Chemistry, University of Edinburgh, West Mains Road, Edinburgh, EH9 3JJ, UK; 2 Medical School, University of Edinburgh, Royal Infirmary Hospital, Little France, Edinburgh, EH16 4SA, UK; 3 Laboratory of Health Effects Research, National Institute for Public Health and the Environment, Bilthoven, The Netherlands

## Abstract

**Background:**

An Aerosol Time-of-Flight Mass Spectrometer (ATOFMS) was used to investigate the size and chemical composition of fine concentrated ambient particles (CAPs) in the size range 0.2–2.6 μm produced by a Versatile Aerosol Concentration Enrichment System (VACES) contained within the Mobile Ambient Particle Concentrator Exposure Laboratory (MAPCEL). The data were collected during a study of human exposure to CAPs, in Edinburgh (UK), in February-March 2004. The air flow prior to, and post, concentration in the VACES was sampled in turn into the ATOFMS, which provides simultaneous size and positive and negative mass spectral data on individual fine particles.

**Results:**

The particle size distribution was unaltered by the concentrator over the size range 0.2–2.6 μm, with an average enrichment factor during this study of ~5 (after dilution of the final air stream). The mass spectra from single particles were objectively grouped into 20 clusters using the multivariate K-means algorithm and then further grouped manually, according to similarity in composition and time sequence, into 8 main clusters. The particle ensemble was dominated by pure and reacted sea salt and other coarse inorganic dusts (as a consequence of the prevailing maritime-source climatology during the study), with relatively minor contributions from carbonaceous and secondary material. Very minor variations in particle composition were noted pre- and post-particle concentration, but overall there was no evidence of any significant change in particle composition.

**Conclusion:**

These results confirm, via single particle analysis, the preservation of the size distribution and chemical composition of fine ambient PM in the size range 0.2–2.6 μm after passage through the VACES concentration instrumentation.

## Background

Epidemiological studies have consistently shown that elevated levels of particulate matter (PM) air pollution are associated with increases in asthma severity and worsening of respiratory ill-health, as well as increased mortality not only from respiratory causes but also from cardiovascular disease [1,2]. The associations are often strongest for the fine (PM_2.5_) fraction [3] which penetrates to the alveoli of the respiratory system [4].

Two of the most important challenges for researchers in this field are to establish (i) the physicochemical properties of the inhaled particles responsible for observed ill-health associations, and (ii) the consequent biological mechanisms of causation. Progress on the former requires enhanced size and chemical characterisation of the ambient particles to which populations or panel members are exposed, whilst progress on the latter requires experiments testing potential causal mechanisms either *in vitro *or, ideally, *in vivo *using model or genuine particulate matter. A major criticism of most mechanistic studies is that the exposure route is artificial and the PM dose is very high. In the last few years, however, instrumentation has been developed that is designed to deliver a continuous flow of air in which the concentration of ambient PM in the sampled air stream is increased in real-time by roughly an order of magnitude [5,6]. The concentrated PM is generically referred to as CAPs (concentrated ambient particles). The advantage of these instruments is that they can provide genuine inhalation exposures, at PM concentrations not much greater than ambient, with the subject of the exposure (animal or human) assessed under controlled conditions. In example studies using human volunteers, exposure to fine or coarse CAPs has been shown to induce mild pulmonary inflammation in healthy adults [7], and to change heart rate variability in the elderly [8] and in asthmatic and healthy younger adults [9].

It is important that particle concentrators do not alter the size distribution (within the fraction being concentrated) or chemical composition of the particles. In the past, particle composition has been checked using off-line chemical analyses of bulk filter-collected samples [5] but this requires a raft of different analytical techniques, is time-consuming, does not provide information on the state of chemical mixing of individual particles and is necessarily time-averaged. Very recently, single particle mass spectrometers have been deployed for more detailed investigation of the effect of concentrators on the ensembles of individual particles. Using an Aerosol Time-of-Flight Mass Spectrometer (ATOFMS) alongside particle sizing instrumentation, Moffet *et al*. [10] showed that the Harvard/USEPA Ambient Particle Concentrator (HAPC) did not induce observable changes to particles in the coarse (PM_2.5–10_) size fraction. At the other end of the size range, Zhao *et al*. [11] used the Rapid Single-particle Mass Spectrometer (RSMS-3) to investigate the chemical composition of 40–640 nm ultrafine particles pre- and post-concentration by the Versatile Aerosol Concentration Enrichment System (VACES) [12,13]. Although small differences in composition were observed (manifested as a shift of 8–10% of particles from one class to another), this was suggested to be due to changes in the composition of the ambient air rather than changes induced by the VACES. Likewise, in a recent deployment of an Aerosol Mass Spectrometer (AMS) with the VACES, only relatively small changes in mass of semi-volatile material (ammonia, nitrate, organics) was observed in post-concentrator particle mass for particles <~1 μm [14].

The Mobile Ambient Particle Concentrator Exposure Laboratory (MAPCEL) used in this work contained a VACES concentrator, with the aim of concentrating particles of diameter <2.5 μm and delivering them directly to an exposure chamber at a flow rate of 50 L min^-1^, suitable for human breathing. In the first exposure study of its kind, the MAPCEL has recently been used to investigate the effects of inhalation of CAPs on systemic inflammation and vascular function in patients with ischemic heart disease [15]. Twelve male patients with stable ischemic heart disease and twelve age-matched non-smoking volunteers were exposed either to CAPs or to filtered air during 2 h of intermittent exercise using a randomized double-blinded cross-over study design during a 4-week period in February 2004. The CAPs were derived from urban background air outside the Royal Infirmary Hospital in Edinburgh, UK (3.22°W 55.95°N). A bus route passed adjacent to the MAPCEL and an arterial city-route was located a few hundred metres away. Vascular function and systemic inflammatory markers were measured 6–8 hours following exposures [15].

The VACES apparatus has not yet been evaluated with a single particle mass spectrometer over its full operational particle size range up to ~2.5 μm (the upper particle size in the Zhao *et al*. study was 0.64 μm [11]), so the opportunity was taken during this exposure study to deploy an ATOFMS to investigate the effect of the enrichment process on particle composition in this specific size fraction. The advantage of the ATOFMS for this work is that its inlet and sizing system is optimised to detect and ablate particles in the size range 0.2–3.2 μm [16], which overlaps well with the upper part of the size range of CAPs produced by the VACES [12]. The ATOFMS instrument was also used to provide data on PM chemical composition during the human exposures, which in turn will be useful to identify dominating sources of PM emissions.

## Instrumentation and methods

**Figure 1:**
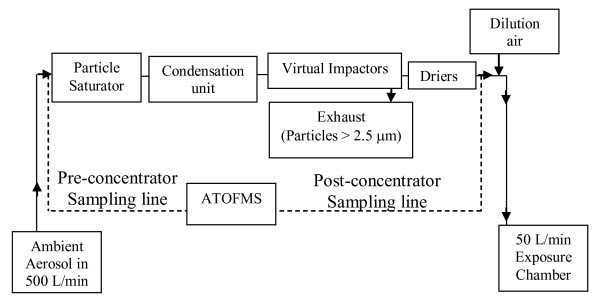
**Schematic diagram depicting the operation of the ambient particle concentrator used in this study**. The dotted lines illustrate sampling points for the ATOFMS. The ATOFMS was connected to each sampling line in turn for approximaetly 40 minutes.

A schematic diagram of the concentrator operational principles is shown in Figure 1. Incoming ambient air at a flow of 500 L min^-1 ^passes through a unit that saturates the air stream with warm water vapour. Incoming particles larger than ~3 μm are lost by impaction and deposition to the walls of the inlet and saturator and do not make it through the system. In the condensation unit each fine particle grows to a few μm in size by water vapour condensation. The number concentration of the enlarged particles is then stepwise increased by taking the minor flow of the air each time it passes through three virtual impactors of ~1.5 μm cut-off in series, (the minor flow contains particles larger in size than the impactor cut-off). The resulting outward flow of 25 L min^-1 ^from the virtual impactors then passes through a number of silica gel dryers which remove the condensed water to return the particles to their original size. To provide a flow of 50 L min^-1 ^required for the human subjects in the body box, the air flow containing the enhanced fine particle concentration was finally diluted with particle-free conditioning air. Particles smaller than ~15–20 nm will not grow in the condensation unit and are lost to the exhaust in the major flow from the impactors.

The ATOFMS was housed inside a transit van adjacent to the MAPCEL and its sample inlet was connected to the airflow in the MAPCEL unit either upstream or downstream of the particle concentration section (as shown in Figure 1) using 1 cm i.d. copper tubing. During the human exposure studies the ATOFMS sampled the ambient air being drawn into the body box. Portable heaters inside the van maintained a constant environment of ~20°C around the ATOMFS instrument. The key operational feature of the ATOFMS is that size and chemical information is obtained simultaneously for each individual particle detected [17,18]. Particles in the sampled air are collimated by a nozzle into a low pressure air-stream and scatter light from two continuous-wave lasers (wavelength 532 nm) spaced a few cm apart. The transit time of a particle between the two laser beams (of the order of 0.5 ms) yields the aerodynamic diameter of the particle, the arrival time of the particle in the subsequent ablation region, and the appropriate delay for firing the frequency-quadrupled Nd:YAG ablation/ionisation laser (266 nm). The resultant positive and negative ions are accelerated into two opposing reflectron time-of-flight mass analysers and detected by microchannel plates, yielding positive and negative ion mass spectra for each ablated particle. The major strength of the ATOFMS is the detailed chemical compositional data provided for each individual particle detected. The major limitations are that only a small proportion of particles in a given air sample are sized and ablated, hence there is uncertainty in scaling up ATOFMS-detected particles to a true particle number, and that because of the intrinsic variability of a laser desorption ionisation process chemical information is not fully quantitative [16,19,20].

The analysis of the particle mass spectra dataset was carried out using the MINITAB statistical package. The principal multivariate technique employed was non-hierarchical K-means clustering (Euclidian distance, centroid linkage) using peak area at each integer *m/z *value as the variables. For inclusion as a variable, the area of each peak had to constitute >0.001 of the total peak area in that spectrum. Correlation, rather than co-variance, between variables was used so that clustering was not dominated by one or two persistently large peak areas in the spectra. Twenty clusters were initially specified to classify the data set. Although this resulted in a number of the clusters containing <1% each of the total number of particles in the whole dataset, it allowed a sufficient number of clusters to be created to describe different types of particle. If subsequent inspection showed that two or more clusters contained particles with similar mass spectra, size distribution and temporal trends, the clusters were combined.

## Results and discussion

### Pre- and post-concentrator characterization of CAPs

Air from the concentrator to the ATOFMS was switched every 40 minutes between the upstream and downstream of the concentrator ("pre-" and "post-concentration") during two sampling periods 11:02–12:40 GMT on 17/03/04 (Study A) and 10:53–12:54 GMT on 18/03/04 (Study B).

When sampling relatively warm and humid air into the ATOFMS there is the possibility that condensation of water vapour onto the particles may occur, as reported in the study by Moffet *et al*. [10] carried out in North Carolina. However, for the duration of the CAPs-ATOFMS study reported here (February and March in Edinburgh), the temperature of the ambient air was below that of the temperature in the MAPCEL and ATOFMS (Table 1) so condensation onto sampled particles is not expected.

**Table 1:** Dates, times and meteorological data during the studies presented.

**Date**	**Sampling time**	**Mean temp (°C)**	**Mean wind direction (°)**	**Mean wind speed (m/s)**	**Mean RH (%)**
02/02/04	7:51:28 – 12:00:32	7.6	228	4.5	81
03/02/04	No ATOFMS data	13	207	10.5	82
04/02/04	7:39:54 – 10:31:07	9.1	230	6.3	78
05/02/04	7:57:09 – 10:31:09	10.6	234	8.5	70
					
09/02/04	8:59:42 – 11:04:09	1.5	255	2.8	72
10/02/04	7:46:12 – 10:53:01	8.7	250	9.7	85
11/02/04	9:55:03 – 12:11:06	8.9	230	2.4	83
12/02/0	7:44:37 – 10:56:50	4.2	242	0.8	97
					
17/02/04	10:53:37 – 12:54:57	5.5	280	1.5	75
18/02/04	11:02:12 – 12:40:46	5.4	223	0.7	71

#### Enrichment factors and effect on size distribution

**Figure 2:**
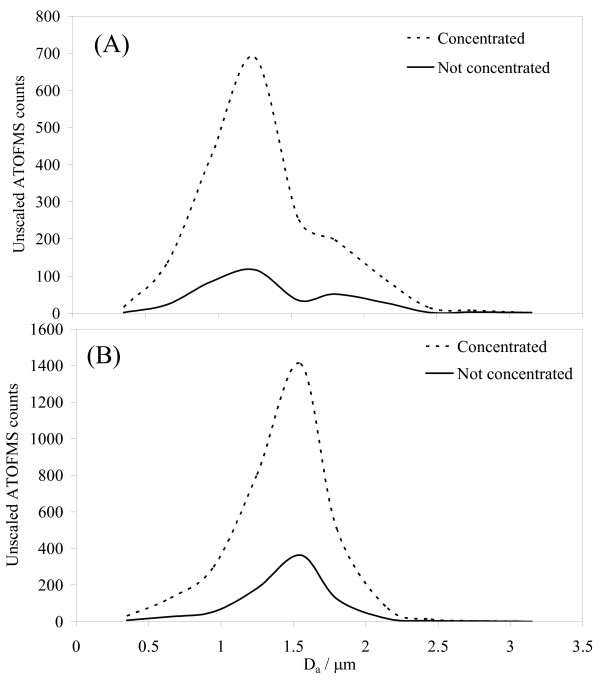
**Pre- and post-concentrator particle size distributions for characterisation studies A and B**. Particle numbers are plotted as a function of geometric mean diameter for the size ranges (in μm): 0.2–0.5, 0.5–0.8, 0.8–1.1, 1.1–1.4, 1.4–1.7, 1.7–1.9, 1.9–2.3, 2.3–2.6, 2.6–2.9 and 2.9–3.2.

The pre- and post-concentration particle size distributions measured by the ATOFMS are shown in Figure 2. Differences in particle size distribution between the two characterisation studies, with modes of 1.2 μm and 1.5 μm respectively, reflects differences in the chemical composition of the particles detected (see below). Although the ATOFMS detects particles with diameter in the range 0.2–3.2 μm, only a proportion of particles are detected, and this proportion varies with particle diameter [16,20]. Thus the curves in Figure 2 are not the true particle size distribution but nevertheless permit comparison of relative numbers pre- and post-concentration. With proper particle number instrumentation it is possible to derive scaling factors for the ATOFMS-derived number concentration as a function of particle diameter [16,20]. No scaling was undertaken in this study but, in principle, the enrichment factor, EF (the ratio of post- and pre-particle concentration) at a given particle size can be derived from a proportional or absolute measure of total particle number. However, it should be recognised that EFs derived from ATOFMS data will be subject to uncertainty introduced by the inevitable instant-by-instant variability in the proportion of particles detected by the ATOFMS.

Table 2 lists the concentrator EFs in this study as a function of particle diameter between 0.2 and 2.6 μm. The EF was essentially constant across this size range, consistent with the data presented by Kim *et al*. [12]. (Quantification of an EF for particles >2.6 μm was not reliable because extremely few particles of this size passed through the VACES). The overall EF integrated over the whole size range 0.2–2.6 μm in both studies was 5, in agreement with the EF range of 4–6 measured concurrently by filter gravimetry for each of the human exposures in the MAPCEL, but somewhat lower than the EF of ~10–30 (varying inversely with virtual impactor minor flow rate) reported by Kim *et al*. [12] (although these authors also observed a decline in EF between 1 and 2 μm). Zhao *et al*. [11], also using only a single-particle mass spectrometer, reported variation in "hit rate enhancement" from ~30 for particles of 18 nm diameter to ~5 at 1 μm. The differences in EF between studies presumably arise from different concentrator operational conditions, including the requirement in this study for final air flow to be diluted to 50 L min^-1 ^for the exercising volunteers. The invariant fractions of sub- and super-micron particles before and after concentration (Table 3) further supports the lack of effect of the concentrator on the particle size distribution.

**Table 2:** Enrichment factors (EF) as a function of particle diameter (*D*_a_) for the two characterization studies A and B.

**Study A**		**Study B**	
	
***D*_*a *_(μm)**	**EF**	***D*_*a *_(μm)**	**EF**
	
0.20 – 0.79	6 ± 2	0.20 – 0.79	5 ± 1
0.80 – 1.39	5 ± 1	0.80 – 1.39	5 ± 1
1.40 – 1.99	5 ± 3	1.40 – 1.99	4 ± 1
2.00 – 2.59	4 ± 1	2.00 – 2.59	5 ± 2

Errors are 95% confidence intervals

**Table 3:** Percentage of sub-micron and super micron particles detected during the two characterisation studies A and B.

	**% Sub-micron particles**	**% Super-micron particles**
**Study A**		
Pre-concentration	32	68
Post-concentration	33	67
**Study B**		
Pre-concentration	11	89
Post-concentration	14	86

#### Effect on composition

As described in the experimental section, the entire dataset of particles hit by the laser (i.e. particles for which mass spectra were obtained) were initially assigned to 20 clusters by K-means clustering. After further manual examination of the individual mass spectra, size distributions and temporal trends, a number of the clusters were combined, yielding a total of eight particle classes within four broad categories of particle composition: sea-salt (pure, mixed and reacted classes), carbonaceous (elemental carbon and mixed organic/elemental carbon classes), dust (CaSO_4 _and Al/Fe/Li classes) and a mixed-KCl class. The evolution of sea-salt particles, from pure through mixed to reacted, is a consequence of the progressive replacement of chloride with nitrate by reaction with HNO_3_[18]. The distinguishing peaks in the mass spectra of particles assigned to each named class included the following:

(i) *pure sea-salt class*, peaks of composition Na_x_Cl_y_^+/- ^only, with no peaks indicating nitrate ions;

(ii) *mixed sea-salt class*, peaks for Na_x_Cl_y_^+/- ^plus also negative ion signals at -46 (NO_2_^-^) and -62 (NO_3_^-^);

(iii) *reacted sea-salt class*, +23 (Na^+^), +39 (NaO^+^), +40 (NaOH^+^), plus nitrate ion signals in the negative spectra, and no peaks corresponding to Cl;

(iv) *elemental carbon class*, peaks of the C_n_^+ ^series but no hydrocarbon signals;

(v) *mixed OC/EC class*, +15 (CH_3_^+^), +27 (C_2_H_3_^+^), and +43 (C_3_H_7_^+^);

(vi) *CaSO*_4 _*dust class*, +40 (Ca^+^), +56 (CaO^+^) and sulphate peaks, -96 (SO_4_^-^) and -97 (HSO_4_^-^), in the negative ion spectrum;

(vii) *Al/Fe/Li dust class*, +27 (Al), +54/56 (Fe^+^), and +7 (Li^+^) and signals in the negative ion spectra for silicates, -60 (SiO_2_^-^) and -76 (SiO_3_^-^), and phosphates, -63 (PO_2_^-^) and -79 (PO_3_^-^);

(viii) *mixed-KCl class*, +39 (K^+^), +113/115 ((KCl)K^+^) and peaks for nitrate and sulphate in the negative ion spectrum indicating presence of secondary inorganic aerosol.

Particles containing CaSO_4 _(gypsum) are readily identified as arising from a construction-type source (construction was taking place at the site), whilst particles in the Al/Fe/Li class are due to a crustal/sand source (nearby roads were having rock-salt and grit applied to them daily).

**Figure 3:**
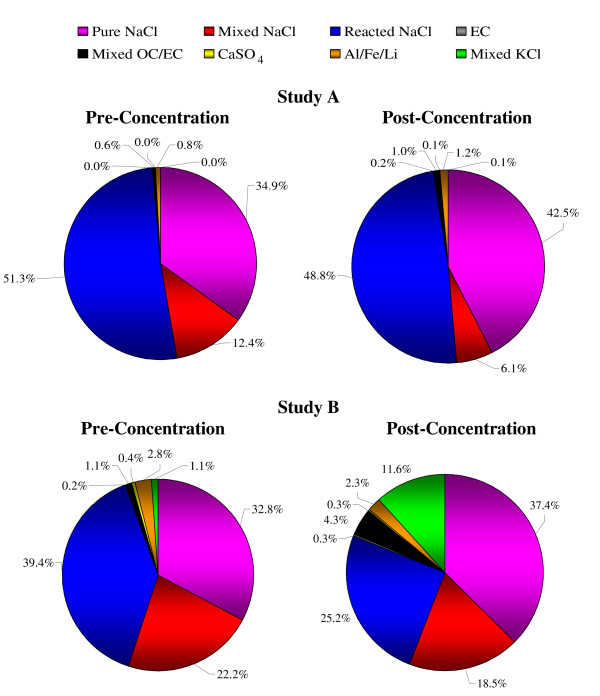
**Pie charts showing the pre- and post-concentrator fractional abundances of the eight main classified particle types for characterization studies A and B**. The particle classes detailed in the legend are plotted clockwise from the top of each pie chart. (Particle diameters in the range 0.2–3.2 μm).

**Figure 4:**
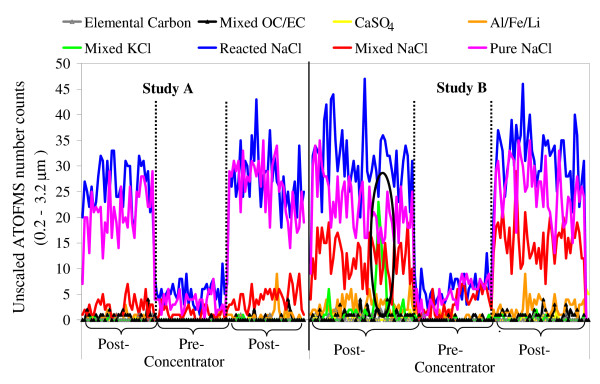
**Time-series of the eight particle cluster types (with diameters from 0.2 – 3.2 μm) identified during characterization studies A and B. **The vertical dotted lines mark the change-over between downstream and upstream sampling. The black ellipse outlines the short episode in which a large number of particles were detected containing KCl and carbonaceous material.

A comparison of the overall particle composition between upstream (pre-concentrator) and downstream (post-concentrator) airflows during the two studies is shown in Figure 3. A time-series of particle class abundances is shown in Figure 4. In general, the majority of particles were classified as pure, mixed or reacted sea-salt. This is not surprising given Edinburgh's maritime climate. Nevertheless the particle chemical composition did differ somewhat between the two studies so the pre- and post-concentrator particle compositions must be compared separately for each study. For example, during characterization study A the particle composition was completely dominated by sea-salt, whereas carbonaceous and dust particles constituted a greater fraction of the particles in characterization study B.

Overall, however, when comparing pre- and post-concentrator particle composition, no significant difference in the proportion of each broad particle type was observed. Close examination of the dataset revealed that the particle type classified as mixed-KCl, which did appear to show a change in abundance between pre- and post-concentration, was in fact only detected during the first session of post-concentrator sampling in the second characterization study (illustrated by the circled region on the time-series in Figure 4). Since this particle type only appeared during one short period of time, it is concluded that this particle type reflects a genuine difference in ambient particle composition at this time rather than a compositional change induced by the particle concentrator. Particles in this class were within the size range 0.4 to 1.9 μm. These particles are likely to have originated from burning of biomass, for which potassium is a known marker [21].

Figure 3 indicates a slight tendency (particularly in the first characterization study) for the proportion of pure sea-salt particles downstream of the concentrator to be enhanced at the expense of mixed sea-salt particles upstream of the concentrator. It is possible to speculate that the condensation of water onto the particles and the subsequent evaporation process causes a slight loss of particulate nitrate as gaseous HNO_3_, in a reverse of the sea-salt aging reaction that occurs in ambient air (described above). The trend is not observed in the second characterization study so there is inconclusive evidence for a concentrator effect with this particle class.

One of the 20 particle clusters originally identified by the K-means process contained particles with mass spectra identical to other particles subsequently classed as Al/Fe/Li dust except that the spectra of the former particles contained more intense high mass negative ion signals for silicates, phosphates and aluminium oxides (e.g. -119 (AlSiO_4_^-^), -122 (AlPO_4_^-^), -140 ((SiO_2_)(HPO_3_)^-^), -179 (AlSi_2_O_6_^-^)) than spectra for the latter, and were only apparent when sampling the post-concentration air flow. The former cluster contained <2.4 % of the total particle dataset, and since there was no actual difference in particle composition, only in intensities of the ion signals, it was concluded that the concentrator had not caused changes to these particles so they were also assigned to the Al/Fe/Li dust class. Although ion intensities in LDI analyses are known to be matrix-dependent [22], the consistent mass spectra overall indicate that there was no difference in the matrices in this instance. A more likely explanation is a straightforward cluster-boundary artefact introduced by the clustering algorithm as has been noted previously by Zhao *et al*. [11] who observed that the ART-2a neural network algorithm split an ensemble of mixed carbonaceous-ammonium nitrate particles into two depending on the intensity of the nitrate signals.

### Chemical characterization of Edinburgh CAPs during human exposure study

**Figure 5:**
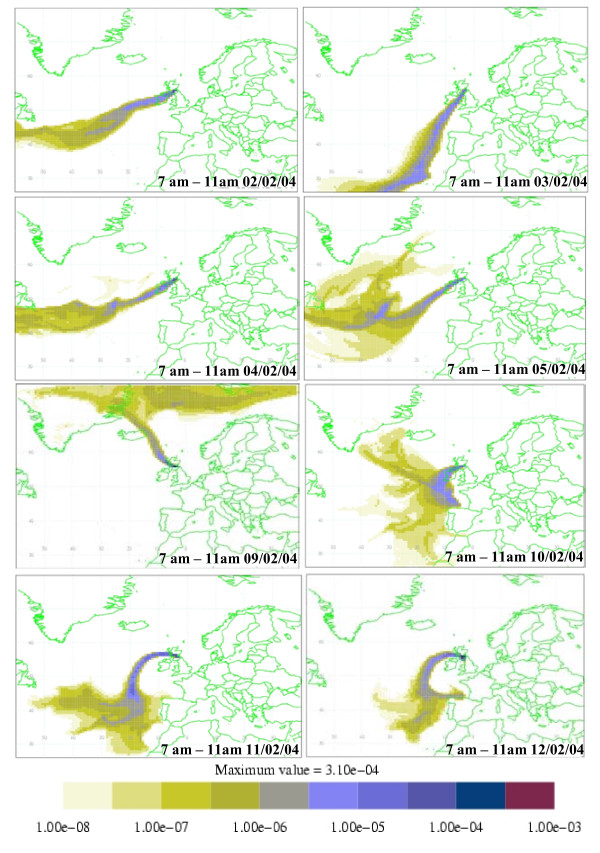
**Five-day back maps for air arriving in the boundary layer in Edinburgh during the CAPs exposure dates/times indicated**. The scale units indicate the relative contribution of the given source area to the air passing over the receptor location at the time of measurement.

The ATOFMS was used to analyse the ambient particular matter in parallel with human CAPs exposures. Details of the ambient conditions are given in Table 1. Air-mass source attribution plots for the 5 days prior to arrival at the sampling location were calculated for each of the exposure periods using the UK Met Office "NAME" model [23]. In all cases air clearly originated either predominantly from the Atlantic or the Arctic with very little contribution from air passing over land apart from final arrival over central Scotland (Figure 5).

**Figure 6:**
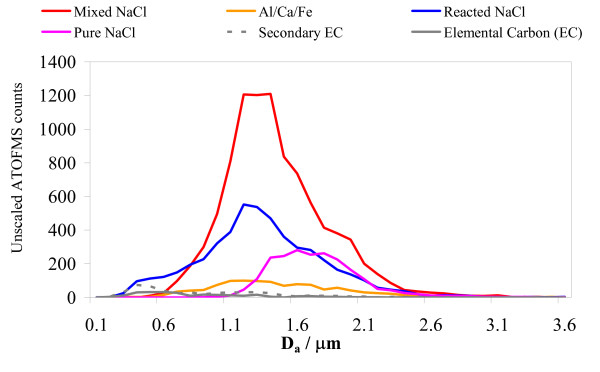
Ambient particle size distributions for each of the six main classified particle types (with diameters from 0.2 – 3.2 μm) detected during the whole period of the CAPs human exposures.

The whole dataset of particles was classified using the methodology previously described. The CaSO_4 _dust class was so infrequent in the exposure study that it was amalgamated with the other dust particle class to form an Al/Fe/Ca dust class. There was no detection of particles corresponding to the previous mixed-KCl class. The time-series of the resulting six particle classes (data not shown) reveals that the composition of the particles remained broadly constant throughout the whole exposure study. Particles classified as one of the sea-salt classes completely dominated the particle types detected, together contributing 90% of all particles detected. This is entirely consistent with the universal marine air-mass source during this study. The sea-salt particles were predominantly in the super-micron size range (Figure 6).

Carbonaceous particles constituted 4% of all particles detected overall and dominated the finer size fraction. These must arise from local traffic and solid-fuel burning sources, given the predominant clean-air source regions. Dust particles contributed the remaining 6% of particles and had a broad monomodal size distribution (Figure 6). There was a slight increase in proportion of both carbonaceous and dust particles in the last couple of days of the exposure sampling period.

A detailed report on the health effects of the CAPs exposures will be presented elsewhere, but in summary, exposure either to the above-described Edinburgh CAPs or to filtered air did not affect vascular function in either patients with stable ischaemic heart disease or age-matched controls [15]. In contrast, a parallel double-blind study of the effects of a similar mass-concentration of diesel exhaust particulates, dominated by carbonaceous and organic particles, demonstrated impairment of both vasomotor and endogenous fibrinolytic vascular function following exposure to particulate [24].

## Conclusion

In conclusion, we find no evidence that the VACES installed in the MAPCEL causes any substantive changes in particle size distribution or individual particle composition during concentration of particles within the size range of 0.2–2.6 μm. Contrasting results in controlled human exposure studies using this apparatus highlight the importance of particle composition in determining the adverse vascular effects of exposure to PM_2.5_. Detailed characterisation of particle exposures, including the use of single-particle mass spectrometry, will be essential in future experimental studies to determine the impact of inhaled environmental particulate matter on public health.

## Competing interests

The authors declare that they have no competing interests.

## Authors' contributions

EJF operated the ATOFMS, collected and analysed the single-particle data and co-wrote the manuscript with MRH, who also contributed to the single-particle data analysis. RJD conceived and organised the single-particle evaluation study. NLM, KD, DEN, PHBF and FRC all participated in the design and implementation of the human exposure study, with PHBF and FRC responsible for implementing CAPs exposures, and NLM, KD and DEN responsible for assessments and interpretation of vascular function data in that study. All authors read, commented on and approved the final manuscript.
